# The Role of Serotonin Transporter in Human Lung Development and in Neonatal Lung Disorders

**DOI:** 10.1155/2017/9064046

**Published:** 2017-02-20

**Authors:** E. C. C. Castro, P. Sen, W. T. Parks, C. Langston, C. Galambos

**Affiliations:** ^1^Department of Pathology & Immunology, Texas Children's Hospital, Baylor College of Medicine, 6621 Fannin St. MC-1195, Houston, TX 77030, USA; ^2^Department of Pediatrics, Baylor College of Medicine, 6621 Fannin St. MC-1195, Houston, TX 77030, USA; ^3^Department of Pathology, Magee Women's Hospital, UPMC, Pittsburgh, PA 15213, USA; ^4^Department of Pathology and Laboratory Medicine, Children's Hospital Colorado, University School of Medicine of Colorado, 13123 East 16th Avenue, P.O. Box 120, Aurora, CO 80045, USA

## Abstract

*Introduction.* Failure of the vascular pulmonary remodeling at birth often manifests as pulmonary hypertension (PHT) and is associated with a variety of neonatal lung disorders including a uniformly fatal developmental disorder known as alveolar capillary dysplasia with misalignment of pulmonary veins (ACD/MPV). Serum serotonin regulation has been linked to pulmonary vascular function and disease, and serotonin transporter (SERT) is thought to be one of the key regulators in these processes. We sought to find evidence of a role that SERT plays in the neonatal respiratory adaptation process and in the pathomechanism of ACD/MPV.* Methods.* We used histology and immunohistochemistry to determine the timetable of SERT protein expression in normal human fetal and postnatal lungs and in cases of newborn and childhood PHT of varied etiology. In addition, we tested for a SERT gene promoter defect in ACD/MPV patients.* Results.* We found that SERT protein expression begins at 30 weeks of gestation, increases to term, and stays high postnatally. ACD/MPV patients had diminished SERT expression without SERT promoter alteration.* Conclusion.* We concluded that SERT/serotonin pathway is crucial in the process of pulmonary vascular remodeling/adaptation at birth and plays a key role in the pathobiology of ACD/MPV.

## 1. Introduction

There is a remarkable change in the structure and function of the pulmonary vasculature during the transition from fetal to extrauterine life. In the fetus, the pulmonary vasculature is a high resistance low flow system, and thus right ventricular output is shunted to the placenta for gas exchange. At birth, even with the first breath, pulmonary vascular resistance falls dramatically, and pulmonary blood flow increases rapidly. Failure of this transition can lead to serious neonatal lung disorders characterized by the clinical syndrome of Persistent Pulmonary Hypertension of the Newborn (PPHN) [1, 2]. A lethal form of PPHN, alveolar capillary dysplasia with misalignment of pulmonary veins (ACD/MPV), is characterized by vascular abnormalities including a dysplastic pulmonary microvascular network with drastically reduced numbers of capillaries that are often markedly expanded and malpositioned, misalignment of pulmonary veins, and abnormally thickened small arteries/arterioles, as well as lobular underdevelopment. Clinically, infants with ACD/MPV commonly present with respiratory failure and treatment-resistant PHT at or a few days after birth. Associated morbid conditions are well-known. A significantly high number (approximately 80%) of the babies with ACD/MPV have gastrointestinal, cardiac, or genitourinary anomalies in addition to the lung disorder [3, 4]. Recently* FOXF1* abnormalities were detected in up to 70% of ACD/MPV cases; however, the exact mechanism through which these genetic abnormalities lead to ACD/MPV is yet to be elucidated [5].

The role of vasoactive mediators in achieving successful pulmonary vascular remodeling at birth has begun to emerge [6]. Bioavailability of the potent vasoconstrictor serotonin has been shown to be predominantly regulated by the serotonin transporter (SERT) in the lung endothelium [7–15]. Abnormal SERT expression can lead to dysfunction of a variety of organs, including the lung [16–21]. Importantly, maternal use of pharmacologic SERT inhibitors (SSRIs) in the third trimester of pregnancy has been linked to the development of PPHN, and altered pulmonary vasculature has been proposed as a possible mechanism [22, 23]. This association indicates a likely critical role for SERT in the normal adaptation of the fetal pulmonary vasculature to extrauterine life. This role of SERT is further substantiated by an animal study in which SSRI exposure in utero induced PH and pulmonary arterial smooth muscle hyperplasia resembling that seen in PPHN [24]. A recent study has shown that serotonin plays a key role in maintaining high levels of pulmonary vascular resistance and that SSRI exposure increases serotonin activity causing pulmonary hypertension in the chronically prepared newborn lamb [25].

Current data suggests that disorders associated with PPHN have an overall mortality rate of 10–20%, although ACD/MPV is uniformly lethal despite the use of inhaled nitric oxide therapy often combined with extracorporeal membrane oxygenation [11, 26]. As the bioavailability of serotonin in the body is primarily regulated by pulmonary SERT [7–15], there is likely a fundamental role for SERT in the regulation of pulmonary vascular tone, particularly for the major changes that occur related to birth.

Thus, we hypothesize that human pulmonary SERT may play a role in the adaptation of fetal pulmonary vasculature to extrauterine life and that abnormalities of SERT activity lead to neonatal pulmonary hypertension. Here we present a study of SERT expression in pre- and postnatal human lungs and in cases of newborn and childhood PHT of varied etiology including ACD/MPV.

## 2. Material and Methods

### 2.1. Tissue Samples

Approval for this study was obtained through review by the University of Pittsburgh and Baylor College of Medicine Institutional Review Boards.

Microarray of pulmonary tissue obtained from multiple autopsy cases was employed to establish the specificity and sensitivity of SERT immunostaining of lung endothelium. Additionally, SERT immunostaining of the vascular endothelial of different organs was similarly assessed.

Physiologic temporal-spatial expression of SERT was studied using lung sections from 30 autopsy cases encompassing a range of ages, including 27 cases of 12, 13, 16, 18, 34, 39 and 40 weeks of gestational age (WGA) and 3 postnatal lungs of ages 1, 5, and 10 years. Cases with significant pulmonary pathology were excluded from this group.

SERT expression in a variety pulmonary hypertensive disorders was studied employing lung sections obtained at autopsy from 29 children with PHT due to ACD/MPV (*n* = 14), congenital alveolar dysplasia (*n* = 1), congenital diaphragmatic hernia (*n* = 5), bronchopulmonary dysplasia (*n* = 3), secondary PHT in congenital heart disease (*n* = 1), and idiopathic primary PHT (*n* = 5). For these 29 cases, patient histories and histologic sections of the lung were reviewed to confirm the initial diagnosis. All ACD/MPV patients were tested for mutation in* FOXF1* and 4 were tested for rearrangements at 16q24 locus. Among the 14 ACD/MPV patients, three had mutations in* FOXF1*. Of the 11 without identified mutation, 4 were further identified with rearrangements at 16q24 locus with deletions identified in three (Table 1).

### 2.2. Immunohistochemistry

**Figure 1:**
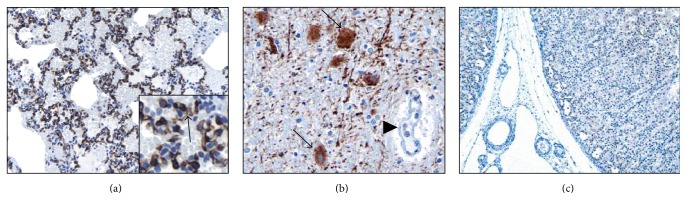
*SERT expression is specific and sensitive to the lung microvasculature* (3,3′-diaminobenzidine, SERT antibody expression in brown). Autopsy tissue microarrays from newborns to 10 years of age showed crisp and diffuse SERT expression (arrow) in lung endothelium ((a) 200x; inset is high magnification 600x), with positive controls ((b) 200x) highlighting numerous serotoninergic neurons (arrows) in the Raphe nucleus (note negative endothelial staining of the adjacent small artery on the right, arrow head); all other organs had negative endothelial cell SERT staining as seen here in the adrenal ((c) 20x, no brown stain noted).

SERT (mouse monoclonal, clone MAB5618; Millipore, Temecula, CA, USA) and CD31 (mouse monoclonal, clone JC70A; Dako, Carpinteria, CA, USA) antibodies were used as follows: after the routine steps of deparaffinization and dehydration SERT antigen was retrieved by steam-heat treatment in 1 mM EDTA + 10 mM Tris buffer at pH 9.0. After incubation of primary SERT antibody for 30 minutes in room temperature at a dilution of 1/4000, diaminobenzidine (DAB) staining was carried out with utilization of the avidin-biotin-peroxidase system. All sections were counterstained with hematoxylin. Diffuse brown membranous endothelial staining was interpreted as positive while absence of brown staining was deemed negative. For positive controls brain sections including the Raphe nucleus (rich in serotoninergic neurons and recommended by manufacturer) were used; adrenal tissue was used as negative control (Figure 1). The ACD cases were always stained in batches containing the positive and negative control as well as the pan-endothelial marker CD31 to assess pulmonary endothelial cell preservation. Diffuse and intense endothelial cell staining was required; otherwise the case was rejected.

### 2.3. SERT Staining Assessment

SERT immunohistochemistry was assessed and recorded as positive and negative. In addition, the terms diffuse and patchy were added for the distribution pattern of the stain.

### 2.4. PCR and Sequencing

The PCR was carried out in a TC-512 thermocycler (Techne, Minneapolis, MN) utilizing 100 ng template DNA using the following protocol: 5 min at 95°C, followed by 35 cycles of 1 min at 95°C, 1 min at 60°C, 2 min at 72°C, and final extension for 10 min at 72°C. The PCR products were purified using a Sephadex G-50 column and sequenced on a ABI 3130XL DNA Genetic Analyzer (Life Technologies, Grand Island, NY) following the manufacturer's protocol. The primers used were as follows: forward-GCCAGCACCTAACCCCTAATG and reverse- GAATACTGGTAGGGTGCAAGGAG.

## 3. Results

### 3.1. Specificity and Sensitivity of SERT to Human Endothelium

The autopsy tissue microarray showed that in normal lungs SERT is expressed specifically in pulmonary endothelial cells with a diffuse crisp brown staining after birth (Figure 1). We also found positive staining in the epithelial cells of gastric glands (4/4 cases), hepatocytes (3/4 cases), and renal tubular epithelium (3/4 cases) and in axons of the brain (3/4 cases) and Schwann cells in the nerve (3/4 cases). One case of 4 showed minimal staining of myocardium and 1/4 showed limited staining in the epithelium of tracheal mucous glands. In no organs, other than the lung, was there positive SERT staining of vascular endothelium.

### 3.2. Expression of SERT in Pre- and Postnatal Human Lungs

**Figure 2:**
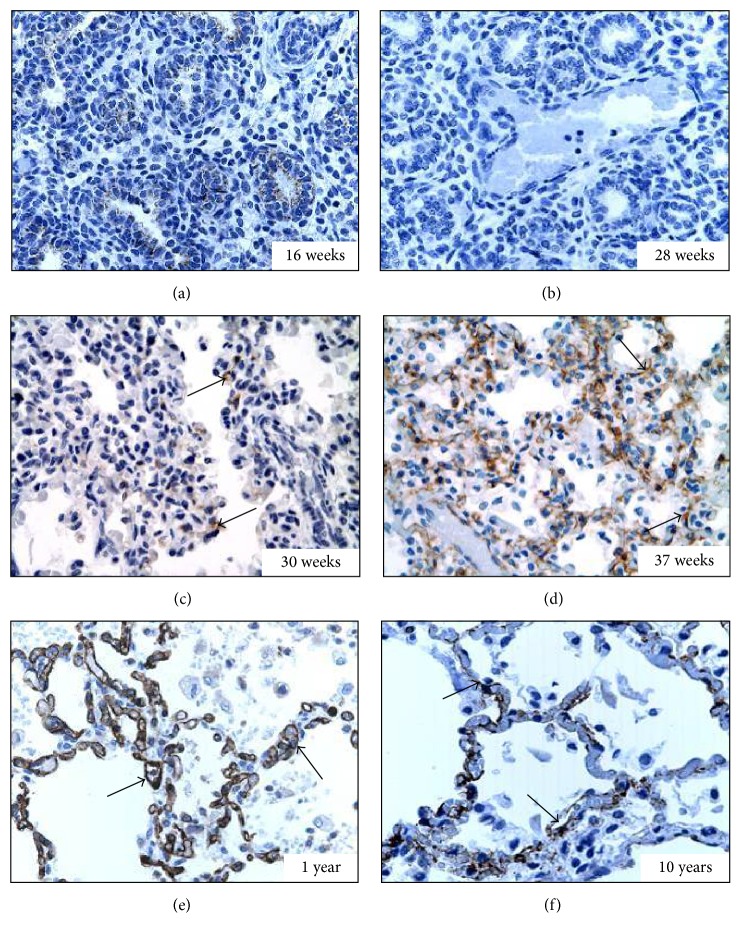
*Expression of SERT in the pulmonary endothelium during development* (3,3′-diaminobenzidine, SERT antibody expression in brown). (a) At 16 WGA (pseudoglandular stage); no SERT expression is detected (400x). (b) At 28 WGA (canalicular stage) no SERT expression is detected (400x). (c) At 30 WGA (saccular stage) patchy capillary SERT staining (arrows) is detected (400x). (d) At 37 WGA (alveolar stage) diffuse and intense microvascular staining of SERT (arrows) is evident (400x). (e) At one year of age SERT staining (arrows) is diffuse and intense (400x). (f) At 10 years of age SERT staining (arrows) remains diffusely present (400x).

Lungs from the antenatal period included all stages of lung development including pseudoglandular (*n* = 3, Figure 2(a)), canalicular (*n* = 13, Figure 2(b)), saccular (*n* = 5, Figure 2(c)), and alveolar (*n* = 6, Figure 2(d)). At very young age (below 29 WGA) there was no SERT staining. Focal capillary endothelial staining (arrows) of low intensity was first noted at 30–34 WGA (Figure 2(c)). By 37 WGA SERT staining became more diffuse (arrows) and intense and at 40 WGA almost all the capillaries were SERT positive when compared to CD31 staining. Postnatally SERT stain remained diffusely positive (arrows) within the pulmonary microvasculature (Figures 2(e) and 2(f)).

### 3.3. Expression of SERT in Lungs with Maladaptive PHT

**Figure 3:**
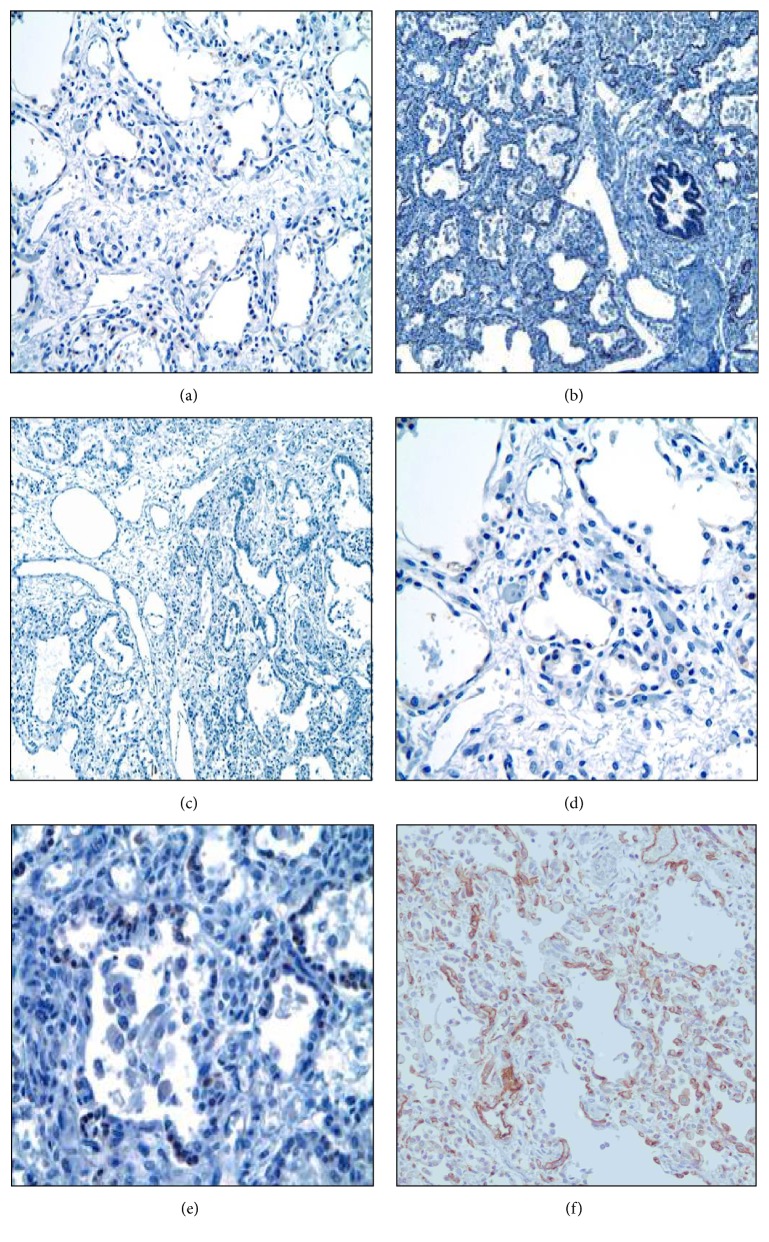
*ACD/MPV pulmonary microvasculature lacks SERT expression* (3,3′-diaminobenzidine, SERT). Representative sections from 5 different ACD cases ((a)–(e)) show absence (no brown stain noted) of SERT staining (200x). (f) Positive control stained with the pan-endothelial marker CD31 (brown stain) to assess pulmonary endothelial cell preservation).

**Figure 4:**
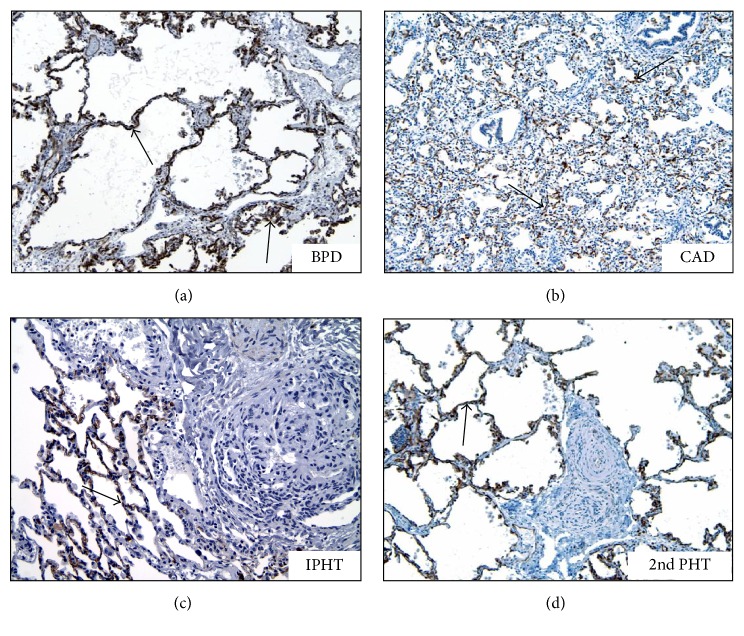
*SERT staining is diffusely present in various pulmonary hypertension disorders other than ACD/MPV* (3,3′-diaminobenzidine, arrows showing SERT antibody expression in brown). BPD: bronchopulmonary dysplasia (200x, arrows), CAD: congenital alveolar dysplasia (100x), IPHN: idiopathic (primary) pulmonary hypertension (200x), and 2nd PHT: secondary PH due to congenital heart disease (200x).

SERT expression was absent or only very minimal in the pulmonary microvasculature in every ACD/MPV case (Figures 3(a)–3(e)). The ACD/MPV cases were always stained in batches containing the positive control stained with the pan-endothelial marker CD31 to assess pulmonary endothelial cell preservation (Figure 3(f)). The pulmonary capillaries from all other cases with PHT of other etiologies showed diffuse and strong SERT staining (Figure 4).

### 3.4. Length Polymorphism (LPR) and Sequence Analysis of SERT (5-HHT) Promoter in the DNA of ACD/MVP Patients

**Figure 5:**
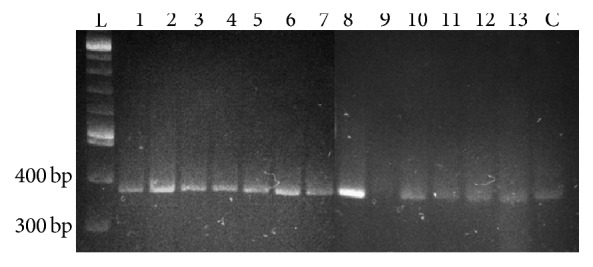
*PCR study of SERT promoter length polymorphism shows the uniform presence of the long allele in 13 ACD/MVP patients* (1–13); L is the DNA ladder and C is the amplicon from a control template.

Several SERT promoter defects have been linked to human disorders [27–36]. Thus, we were interested in knowing if such genetic abnormality might play a role in the regulation of SERT expression in ACD/MVP patients. The PCR result with ACD/MPV DNA samples showed an amplicon of uniform length corresponding to the long (L, normal) allele of the length polymorphic region (LPR) of the SERT promoter. The presence of only one band indicates that these patients were all homozygous for that allele (Figure 5). Sequence analysis of all the amplicons showed perfect homology with the ref seq (NM_001045.4) in the GenBank (data not shown). The ACD/MPV cases included patients with and without identified* FOXF1* abnormalities (Table 1).

## 4. Discussion

We found that SERT is specifically expressed in the microvasculature of the developing human lung beginning at about 30 WGA and that SERT expression increases near birth. SERT expression in the pulmonary microvasculature was absent in all 14 ACD/MPV patients tested.

We did not identify any decrease in SERT expression in the lungs of neonates and young children with PHT due to other causes. In addition, SERT promoter length polymorphism in all ACD/MPV patients was without abnormality.

Our study is the first direct evidence of a functional abnormality of the maldeveloped pulmonary microvasculature of ACD/MPV. In this uniformly lethal condition, death is associated with treatment-resistant PHT, suggesting functionally altered pulmonary vasculature [3]. In the adult population, decreased SERT activity in the pulmonary vasculature either drug-induced, genotypical, or idiopathic has been implicated in the development of pulmonary arterial hypertension [37–39]. The pulmonary microvasculature of ACD/MPV patients is markedly and uniformly depleted of SERT expression, suggesting that disruption of SERT/serotonin pathway is a key component of the pathomechanism of ACD/MPV. Because of the rarity of this disorder, we were unable to measure serotonin levels in the ACD/MPV patient group. Based on recent evidence that serotonin is exclusively regulated by lung SERT activity and that increased serotonin levels lead to increased pulmonary vascular resistance and PHT [25], it seems likely that decreased SERT activity leads to increased serotonin levels in ACD/MPV patients. Age-matched control studies of serum serotonin measurements might provide direct evidence for diminished SERT function in patients with ACD/MPV.

The finding that the expression of SERT peaks at or near birth points to a regulatory role for SERT in governing normal pulmonary vascular growth and remodeling, particularly at the critical period near birth. SERT expression begins in the late saccular stage (30 WGA) while the morphologic changes of ACD/MPV begin earlier, likely before the canalicular stage (starting at ~20 WGA) [40]. Therefore the SERT expression defect may be a consequence of an earlier as yet unknown primary event. The regular association of* FOXF1* abnormalities with ACD/MPV suggests that detailed studies targeting the relationship between* FOXF1* and SERT may provide valuable data regarding the pathomechanism of this uniformly fatal neonatal lung disorder.

Due to the rarity of ACD/MPV and because it has largely been defined histologically with most diagnoses made at autopsy examination, it has been very challenging to identify cases in which tissue appropriate for complex molecular studies can be obtained [41–43]. There have been only rare cases in which fresh tissues have been obtained for genetic or complex molecular studies involving RNA analyses. With increasing awareness of this disorder, there have been rare cases in which antemortem diagnosis and sample acquisition have become possible, but for the most part formalin fixed and paraffin embedded tissues have been those available to study this disease. With this in mind we designed our immunohistochemistry study very cautiously to ensure accuracy and reproducibility. Positive and negative SERT controls (Figure 1) with positive pan-endothelial marker (CD31) controls (Figure 3(f)) were stained in batch with the patients' slides and blinded for the investigators' assessment. Because we found that, among 26 patients with PH, only the ACD/MPV patients had negative or very minimal SERT staining, we are confident that our findings are reliable even without additional RNA studies. RNA studies, perhaps using rare and fortuitously obtained tissue from diagnostic lung biopsies, would be needed to confirm this data.

The genetics of SERT promoter regulation is complex and several abnormalities have been described that can lead to a decrease in SERT protein expression [27–36]. We attempted to link decreased SERT expression to SERT promoter length polymorphism (SERT-LP), which has been shown to play a role in the pathogenesis of inflammatory bowel disease [29] and autism [41]. A 43-bp deletion or insertion, located within the SERT promoter, results in a short or long allele and short allele has been proposed to reduce SERT expression [31, 32]. Because all our tested subjects were homozygous for the L allele we concluded that SERT-LP was not responsible for the decreased SERT activity in ACD/MPV patients. While we have no data to suggest that the SERT gene is abnormal in concert with the* FOXF1* in ACD/MPV patients, the identification of genetic abnormalities in the SERT gene (either in the coding or in the regulatory regions) could provide a potential explanation for the diminished SERT protein expression.

Most pediatric pathologists, radiologists, and neonatologists consider ACD/MPV to be a difficult to diagnose neonatal lung disease. Our study suggests that absence of SERT immunostaining in the pulmonary vasculature in infants and beyond is an excellent surrogate marker for this disorder, currently diagnosed only by microscopic examination of the lung with hematoxylin and eosin staining [3]. The uniform absence of SERT immunostaining in ACD/MPV could become a powerful diagnostic tool for ACD/MPV in pediatric pathology practice. Additionally, imaging of altered SERT expression in patients with CNS disease has been an active area of medical diagnostic practice and research and thus several radiotracers that specifically bind SERT have been developed. Proton Emission Tomography (PET) and Single Proton Emission Computer Tomography (SPECT) imaging studies have shown that SERT-radiotracers bind not only to SERT in the CNS, but also to lung endothelial cells, apparently with high affinity [44–50]. SERT-radiotracers have been tested in children and found to be safe [47]. In infants with ACD/MPV, without SERT expression as our data suggest, an imaging study such as PET/SPECT utilizing SERT-radiotracer could be a novel and powerful diagnostic method for ACD/MPV. Significantly, SERT-radiotracers are currently available and, with our data showing that ACD/MPV lungs lack SERT expression, this imaging method could theoretically be clinically implemented immediately. The availability of a new diagnostic imaging study for ACD/MPV could make invasive neonatal lung biopsy unnecessary.

Because diminished SERT expression was unique to ACD/MPV patients with all other patients with neonatal or childhood PH diseases having normal SERT expression, our study provides evidence of a role for SERT-serotonin regulation in the pathogenesis of ACD/MPV. Since the precise mechanism leading to ACD/MPV is unknown, our findings could lead to focused studies to test SERT-relevant pathways [51] in ACD/MPV patients and possibly animal models [52, 53]. Serotonin has been shown to upregulate Rho-kinase signaling which leads to pulmonary vascular smooth muscle proliferation and pulmonary hypertension [54]. SERT-mediated serotonin uptake is regulated by a number of signaling molecules including p38-MAPK [55] and Syn-1A [56]. In the future, identification of SERT/serotonin pathway defects could also direct the development of new therapeutic strategies targeting their related functions in newborns with ACD/MPV. Controlling/modulating SERT expression and/or serotonin levels could potentially slow the progression of pulmonary hypertension in such infants.

We conclude that abnormalities in pulmonary SERT protein expression contribute to the pathomechanism of ACD/MPV. We speculate that the absence of SERT activity leading to high levels of serotonin results in the maintenance of high pulmonary vascular resistance and low pulmonary blood flow seen during fetal life and contributes to the characteristic vascular abnormalities and pulmonary hypertension of ACD/MPV.

## Figures and Tables

**Table 1:** ACD/MVP patient population with testing for mutation and/or deletions in *FOXF1*. Cases not tested for deletions typically had fragmented and/or poorly preserved DNA making testing problematic.

Serial number	Gender	Survival in days	Gestation	Weight	Diagnosis	FOXF1 mutation	FOXF1 deletion	Other associated anomalies
1	F	5	39-5/7	3850	Autopsy	NM	NT	Coarctation of aorta; ventricular septal defect; mitral valve malformation; tricuspid valve malformation; malformed atrial septum

2	M	22	39	3240	Autopsy	NM	NT	Bilateral hydronephrosis secondary to posterior urethral valves

3	F	33	36-1/2	3120	Autopsy	NM	NT	None

4	M	12	37	N/A	Autopsy	NM	NT	Right lung with two lobes

5	M	4	38	3295	Autopsy	NM	NT	Marked hypertrophy of urinary bladder wall; bilaterally dilated ureters and renal pelves

6	F	9	40	4180	Autopsy	NM	NT	Butterfly vertebra; imperforate anus; abnormal pulmonary lobar configuration

7	F	30	41-2/7	3595	Autopsy	NM	NT	Small thymus with adrenal glands; single umbilical artery

8	M	47	40	3025	Autopsy	NM	ND	Bilateral ureteropelvic junction obstruction; hydronephrosis

9	M	10	32	2470	Autopsy	c.225C>A; p.Tyr75^*∗*^		Bilateral bilobed lungs; malrotation of the intestine; bilateral hydronephrosis and hydroureters; annular pancreas and gastroduodenal stenosis

10	M	0.5	32	2435	Autopsy	c.850dupT; p.Tyr284Leufs^*∗*^11		Lymphangiectasia; renal cortical cysts; pneumothorax; renal obstructive dysplasia

11	M	46	40	5057	Autopsy	c.1138T>C; p.380Argext^*∗*^73		Malrotation of the colon with Meckel's diverticulum

12	F	13	40	3600	Biopsy	NM	Upstream	None

13	F	25	N/A	3676	Biopsy	NM	Genic	Abnormal lobation

14	M	40	38	2900	Autopsy	NM	Genic	Ventricular septal defect; duodenal atresia; annular pancreas; imperforate anus; hydronephrosis; tetralogy of Fallot

NM, no mutation; NT, not tested; ND, tested no deletion; Genic, deletions involving *FOXF1* sequences; Upstream, deletions involving upstream sequences of *FOXF1* not involving the gene itself.
